# The Blood Lactate Increase in High Intensity Exercise Is Depressed by *Acanthopanax sieboldianus*

**DOI:** 10.3390/nu5104134

**Published:** 2013-10-16

**Authors:** Morimasa Kato, Shizue Kurakane, Atsuyoshi Nishina, Jaeyoung Park, Hyukki Chang

**Affiliations:** 1 Department of Health and Nutrition, Yonezawa Women’s Junior College of Yamagata Prefecture, Yonezawa 992-0025, Japan; E-Mails: m-kato@yone.ac.jp (M.K.); kurakane@yone.ac.jp (S.K.); nishina.atsuyoshi@nihon-u.ac.jp (A.N.); 2 Department of Sport, Kyungil University, Gyeongsan, Gteongbuk 712-701, Korea; E-Mail: sports@kiu.ac.kr; 3 Department of Human Movement Science, Seoul Women’s University, 621 Hwarangro, Nowon-Gu, Seoul 139-774, Korea

**Keywords:** *Acanthopanax sieboldianus*, running, swimming, blood lactate, fatigue

## Abstract

This study investigates the anti-fatigue effects of *Acanthopanax sieboldianus* (*A. sieboldianus*) at various exercise intensities. Two experiments were conducted in 18 Sprague-Dawley rats. In Experiment 1, a three-stage increment test (15 m/min for 5 min, and 20 m/min for 5 min and 25 m/min for 10 min) was performed using a treadmill. In Experiment 2, a 10-min swimming test was conducted. Blood samples were extracted from each rat before, during and after the exercises and the blood concentrations of lactate and glucose measured. In both experiments, water (control) or *A. sieboldianus* solution (ASS) was administered orally using a zonde 30 min before the exercise. In the swimming test, ASS administration significantly decreased the blood lactate level measured at the end of the exercise and 5 min post-exercise relative to the water group, although the two groups did not differ significantly in the treadmill test. Our study demonstrates that a single oral administration of *A. sieboldianus* prior to high-intensity exercise significantly decreases the blood lactate concentration suggesting that *A. sieboldianus* has an intrinsic anti-fatigue effect.

## 1. Introduction

Previous research has shown that regular physical activity and proper diet are important not only for preventing lifestyle-related diseases but also for promoting and maintaining one’s health [1,2,3]. In recent years, dietary supplements have attracted interest in the expectation that the individual or synergistic effects of their components might alleviate social health problems such as metabolic syndromes and stress-induced psychiatric disorders. Previous studies have shown that *Acanthopanax sieboldianus* (*A. sieboldianus*), a member of the *Araliaceae* family, has antioxidant effects and suppresses the postprandial rise in the blood glucose levels [4,5].

*A. senticosus* Harms (Siberian ginseng) is another member of the *Araliaceae* family, and has long been used as a nourishing tonic. Ingestion of *A. senticosus* Harms influences the central nervous system, causing euphoria, increasing alertness, and improving concentration levels [6]. Additionally, *A. senticosus* Harms has been reported to exert anti-stress effects in rats subjected to water immersion restraint stress and has also been reported to prevent gastric ulcers [7]. A common pathway involves the activation of the brain monoamine and gamma-aminobutyric acid (GABA) system, which activates the metabolism of dopamine, noradrenaline, and serotonin in the cortex and the hypothalamus. These neurotransmitters are also closely related to exercise performance. Accordingly, a recent study in which mice were subjected to swimming exercise reported that ingestion of *A. senticosus* extracts enhanced the animals’ endurance capacity, which suggests that this substance also exerts an anti-fatigue effect during exercise [8].

On the basis of these findings, we proposed a hypothesis that *A. sieboldianus* also exerts an anti-fatigue effect during exercise. It is essential to consider the relationship between exercise and nutritional intake specifically the different physiological responses induced by exercise at various intensities [9]. The upper limit of the workload that can be sustained by aerobic metabolism is known as the Anaerobic Threshold (AT), and the responses of hormonal and ventilation parameters during exercise change at the AT boundary [10,11]. In this study, we performed an incremental treadmill running test at different levels of exercise intensity, including intensities above and below the anaerobic threshold and a swimming test to examine the effects of *A. sieboldianus* intake on the changes in the blood lactate and glucose concentrations during and after exercise.

## 2. Experimental Section

### 2.1. Animals

Adult male Sprague-Dawley rats (*n* = 18) weighing between 230 and 260 g were obtained from CLEA, Japan, Inc. (Tokyo, Japan). All animals were housed in group cages illuminated from 08:00 to 20:00 (12:12-h cycle) at a breeding-appropriate temperature between 21 and 23 °C. Throughout the experiment, the animals received commercial rodent chow CE2 (CLEA Japan Inc., Tokyo, Japan) and water *ad libitum*. All rats were housed under laboratory conditions for 1 week for environmental adaptation. All procedures were performed in accordance with the Institutional Guidelines for Animal Care as specified by the National Institutes of Health Guidelines for the Care and Use of Laboratory Animals. The use of animals was reviewed and approved by the Animal Care Review Committee at Yonezawa Women’s Junior College of Yamagata Prefecture. The habituation protocol was performed to acclimate the rats to running on the treadmill by exercising them on the treadmill for 30 min 5 times a week, with a gradual increase in velocity (from 10 m/min initially to 25 m/min by the end) to allow adaptation. At the end of the treadmill running habituation, a silicone catheter was inserted into each rat’s jugular vein to allow blood sampling during exercise, as previously described [12,13]. Both treadmill habituation and sampling were performed between 09:00 and 12:00. The rats were weighed before and every day after treadmill habituation, and all experiments were performed after every animal had recovered its full pre-habituation body weight. Preliminary experiments indicated that after treadmill habituation, the subjects’ blood lactate levels did not change from their resting levels when the animals were placed on the treadmill. This study did not include a non-exercise group as a control for the changes in the blood lactate level during exercise.

### 2.2. Experiment 1: Treadmill Running Test Protocol

Twelve hours before the experiment, the rats were fasted by removing the chow to minimize the effect of food consumption just prior to the measurements on the blood glucose level. In this experiment, the rats (*n* = 6) were randomly assigned to receive water or *A. sieboldianus* solution (ASS) via zonde on the first day of the experiment and the alternative treatment on the second day. Thirty min after administration of water or ASS (0.15 g/kg, 1 mL), each rat was forced to run a treadmill program with an incrementally increasing speed (15 to 20 to 25 m/min) for a total of 20 min once a day for 2 days. The speed equivalent to the AT in rats is approximately 20 m/min [13]. In this experiment, the rats ran at 20 or 25 m/min for a total of 10 min. This intensity is equivalent to approximately 60% to 70% of maximum oxygen consumption (VO_2max_). At various time-points (pre-exercise, 5, 10, 15, and 20 min into the exercise, and after 5 min of recovery), 10-µL venous blood samples were taken for measurement of lactate and glucose. After the experiment, the rats were returned to their cages.

### 2.3. Experiment 2: Swimming Exercise Test Protocol

The rats were divided into a water-ingestion group (*n* = 6) and an ASS-ingestion group (*n* = 6). The rats in each group were forced to swim in a round-shaped pool filled with tap water under the following conditions: duration = 10 min, temperature = 35–37 °C, diameter = 46 cm, depth = 40 cm. At the end of the 10-min swimming stress protocol, venous blood samples were taken from the jugular catheters while the rats were still in the pool. The rats were then dried and returned to their cages. Blood samples for measurement of lactate and glucose were obtained at post 5 and 10 min after the rats were returned to their cages.

### 2.4. Lactate and Glucose

The lactate (Lactate pro, Arkray, Kyoto, Japan) and glucose (Glutestace GT-1640, Arkray, Kyoto, Japan) concentrations of the whole blood samples were measured using the indicated measurement equipments. The intra-assay coefficients of variance for the blood lactate and glucose concentrations were 3.45% and 3.62% and the inter-assay coefficients of variance were 3.98% and 3.13%, respectively, in this study.

The blood samples were taken before, during (5, 10, 15, and 20 min into the exercise), and 5 min after the treadmill exercise, and before, during (10 min into the exercise), and 5 and 10 min after the swimming exercise. At least 10 µL of blood was taken from the jugular vein catheter before each increasing in the treadmill velocity for measurement of the blood lactate and glucose levels. In addition, the figures of blood lactate and glucose concentrations at each time-point and the time of measurement were plotted and the AUC (Area Under the Curve) values calculated using waveform analysis software (BIMUTAS II. Kissei Comtec, Nagano, Japan) for quantification of the total volume.

### 2.5. Statistical Analysis

All of the data are represented as the mean ± standard error of the mean (SEM). The effects of the variables were analyzed with a 2 × 2 repeated-measures analysis of variance (ANOVA) using the IBM SPSS 20 statistical package. If significant effects or interactions were confirmed, the Tukey post-hoc test was used to determine the significant differences. In experiment 1, treadmill habituation was necessary to train the rats to run on treadmill, which would have made it difficult and time-consuming to use separate ASS and control groups in a crossover design and the two groups were compared using paired *t*-tests. As experiment 2 involved only the first swimming experience of all of the rats, two different groups were set up and evaluated. Therefore, the Mann-Whitney U test, an unpaired test, was used for analysis. In addition, the effect size and power were calculated for each statistic. Statistical significance was accepted at the level of *p* < 0.05.

## 3. Results

### 3.1. Incremental Treadmill Test—Blood Lactate

The results of 2-way ANOVA were (*F*(1,34) = 0.33, *p* = 0.57, ES(*f*) = 0.10, power = 0.13) for the condition (ASS *vs.* water), (*F*(5,34) = 4.71, *p* < 0.01, ES(*f*) = 0.83, power = 1.00) for the time course, and (*F*(5,34) = 0.56, *p* = 0.71, ES(*f*) = 0.29, power = 0.39) for the condition × time course, indicating that the blood lactate level changed significantly over the time course.

**Figure 1:**
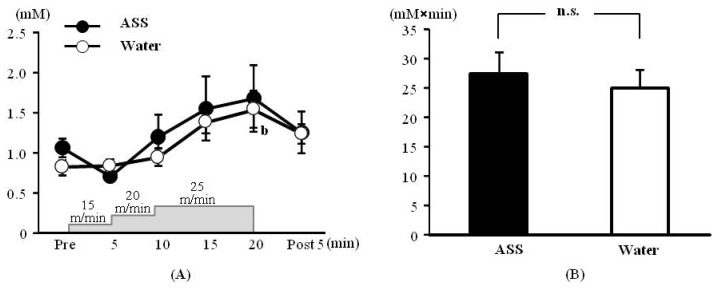
Changes in the blood lactate on incremental treadmill exercise. (**A**) time course change, *x* axis: elapsed time on running, *y* axis: blood lactate concentration (**B**) area under the curve of blood lactate acid, *x* axis; group, *y* axis: total volume of blood lactate. Values are expressed as mean ± SE. b: significant difference from pre (*p* < 0.01). n.s.: no significant differences.

The blood lactate concentrations after administration of ASS were 1.06 ± 0.12 mM at rest, 0.71 ± 0.01 mM at 5 min after the start of exercise, 1.20 ± 0.27 mM at 10 min after the start of exercise, 1.55 ± 0.40 mM at 15 min after the start of exercise, 1.68 ± 0.41 mM at 20 min after the start of exercise and 1.25 ± 0.26 mM at 5 min post-exercise. There was no significant change over the time course (Figure 1A). The values after administration of water were 0.82 ± 0.07 mM at rest, 0.83 ± 0.09 mM at 5 min after the start of exercise, 0.94 ± 0.08 mM at 10 min after the start of exercise, 1.38 ± 0.14 mM at 15 min after the start of exercise, 1.54 ± 0.23 mM at 20 min after the start of exercise and 1.23 ± 0.12 mM at 5 min post exercise. The blood lactate concentration was significantly higher 20 min after the start of exercise than at rest (Figure 1A).

The AUC did not differ significantly between the ASS and control conditions (ASS: 27.4 ± 3.7 mM × min, control: 25.2 ± 2.9 mM × min, *p* = 0.528, ES(*d*) = 0.69, power = 0.28) (Figure 1B).

### 3.2. Incremental Treadmill Running Test—Blood Glucose

The results of 2-way ANOVA were (*F*(1,31) = 0.20, *p* = 0.66, ES(*f*) = 0.08, power = 0.08) for the condition (ASS *vs.* water), (*F*(5,29) = 3.53, *p* < 0.05, ES(*f*) = 0.78, power = 1.00) for the time course, and (*F*(5,29) = 0.46, *p* = 0.80, ES(*f*) = 0.28, power = 0.38) for condition × time course. No significant main effect or interaction was observed.

**Figure 2:**
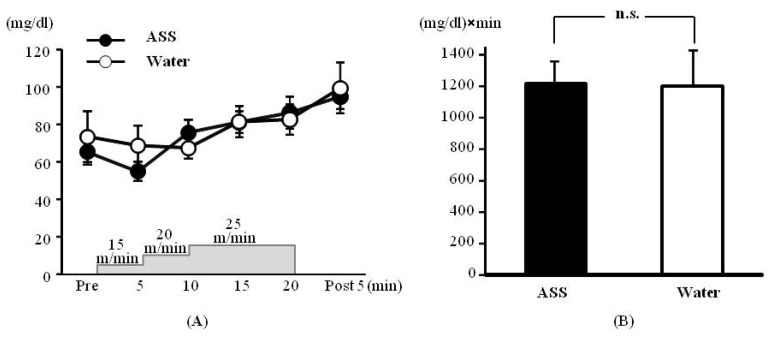
Changes in the blood glucose on incremental treadmill exercise. (**A**) time course change, *x* axis: elapsed time on running, *y* axis: blood glucose concentration (**B**) area under the curve of blood lactate acid, *x* axis; group, *y* axis: total volume of blood glucose. Values are expressed as mean ± SE. n.s.: no significant differences.

The blood glucose concentrations after administration of ASS were 65.33 ± 6.67 mg/dL at rest, 55.00 ± 5.00 mg/dL at 5 min after the start of exercise, 75.75 ± 6.86 mg/dL at 10 min after the start of exercise, 81.25 ± 5.71 mg/dL at 15 min after the start of exercise, 86.25 ± 8.59 mg/dL at 20 min after the start of exercise, and 94.75 ± 6.34 mg/dL at 5 min post-exercise. There was no significant change over the time course (Figure 2A). The values after administration of water were 73.50 ± 13.40 mg/dL at rest, 68.67 ± 10.68 mg/dL at 5 min after the start of exercise, 67.40 ± 5.94 mg/dL at 10 min after the start of exercise, 81.40 ± 8.49 mg/dL at 15 min after the start of exercise, 82.60 ± 8.20 mg/dL at 20 min after the start of exercise, and 99.50 ± 13.50 mg/dL at 5 min post-exercise. There was no significant change over in the time course (Figure 2A).

The AUC did not differ significantly between the ASS and control conditions (ASS: 1233.1 ± 143.9 (mg/dL) × min, control: 1204 ± 225.3 (mg/dL) × min, *p* = 0.892, ES (*d*) = 0.23, power = 0.07) (Figure 2B).

### 3.3. Swimming Exercise Test—Blood Lactate Acid

The results of 2-way ANOVA were (*F*(1,8) = 0.54, *p* < 0.05, ES(*f*) = 0.59, power = 1.00) for the condition (ASS *vs.* water) (*F*(3,24) = 13.17, *p* < 0.01, ES(*f*) = 1.28, power = 1.00) for the time course, and (*F*(3,24) = 2.47, *p* = 0.09, ES (*f*) = 0.56, power = 0.88) for the condition × time course. Significant main effects were observed for both variables.

**Figure 3:**
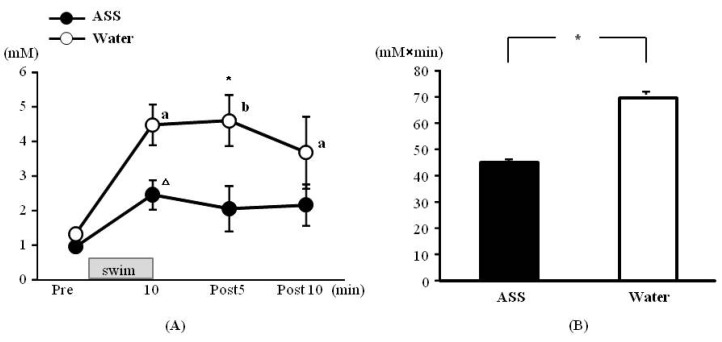
Changes in the blood lactate on swim exercise. (**A**) time course change, *x* axis: elapsed time on swimming, *y* axis: blood lactate concentration (**B**) area under the curve of blood lactate acid, *x* axis; group, *y* axis: total volume of blood lactate. Values are expressed as mean ± SE. a: significant difference from rest (*p* < 0.05). b: significant difference from rest (*p* < 0.01). Δ: *p* = 0.053. *: significant difference between ASS and control (*p* < 0.05).

The blood lactate concentrations in the ASS group were 0.96 ± 0.12 mM at rest, 2.46 ± 0.42 mM at 10 min after the start of exercise, 2.06 ± 0.66 mM at 5 min of post-exercise and 2.16 ± 0.61 mM at 10 min post-exercise. There was no significant change over in the time course (Figure 3A). The values in the water group were 1.32 ± 0.10 mM at rest, 4.48 ± 0.60 mM at 10 min after the start of exercise, 4.60 ± 0.73 mM at 5 min of post-exercise, and 3.68 ± 1.04 mM at 10 min post-exercise. The blood lactate levels measured from 10 min after the start of exercise to 10 min post-exercise were significantly higher than the resting level (Figure 3A). Comparison between the two conditions at 5 min post-exercise revealed that the lactate level was significantly lower in the ASS group than in the control group (Figure 3A).

The AUC was significantly lower in the ASS group than in the control group (ASS: 45.2 ± 8.7 mM × min, control: 70.8 ± 8.5 mM × min) (Figure 3B).

### 3.4. Swimming Exercise Test: Blood Glucose

The results of 2-way ANOVA were (*F*(1,10) = 0.52, *p* = 0.52, ES(*f*) = 0.23, power = 0.34) for the condition (ASS *vs.* water), (*F*(3,30) = 5.81, *p* < 0.01, ES(*f*) = 0.76, power = 0.99) for the time course, and (*F*(3,30) = 0.08, *p* = 0.97, ES(*f*) = 0.09, power = 0.07) for the condition × time course. A significant main effect of the time course was observed.

**Figure 4:**
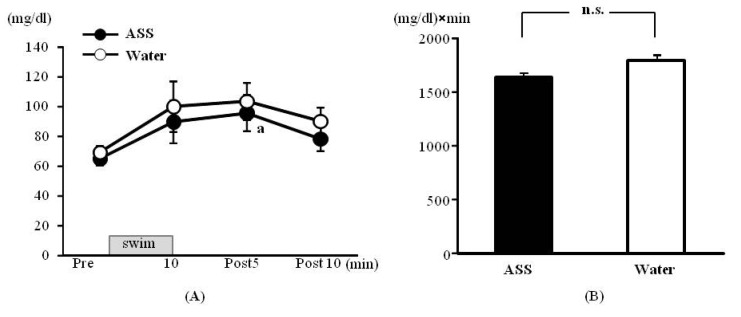
Changes in the blood glucose on swim exercise. (**A**) time course change, *x* axis: elapsed time on swimming, *y* axis: blood glucose concentration (**B**) area under the curve of blood lactate acid, *x* axis: group, *y* axis: total volume of blood glucose. Values are expressed as mean ± SE. a: significant difference from rest (*p* < 0.05). n.s.: no significant differences.

The blood glucose levels in the ASS group were 65.00 ± 4.48 mg/dL at rest, 89.67 ± 14.20 mg/dL at 10 min after the start of exercise, 95.50 ± 12.09 mg/dL at 5 min post-exercise, and 78.17 ± 8.02 mg/dL at 10 min post-exercise. The value was significantly higher 5 min post-exercise than at rest (Figreu 4A). The blood glucose levels in the control group were 69.17 ± 4.48 mg/dL at rest, 99.83 ± 16.83 mg/dL at 10 min after the start of exercise, 103.33 ± 12.59 mg/dL at 5 min post-exercise and 90.00 ± 9.27 mg/dL at 10 min post exercise. There was no significant change over the time course (Figure 4A).

The AUC did not differ significantly between the ASS and control groups (ASS: 1670.7 ± 188.1 (mg/dL) × min, control: 1836.3 ± 174.4 (mg/dL) × min, *p* = 0.423, ES(*d*) = 0.37, power = 0.09) (Figure 4B).

## 4. Discussion

We investigated the anti-fatigue effects of *A. sieboldianus* by subjecting rats to exercise at several levels of intensity and found for the first time that *A. sieboldianus* intake before high-intensity exercise decreases the blood level of lactate, a substance generated during physical activity. This finding suggests that *A. sieboldianus* intake could help to alleviate fatigue during high-intensity exercise. Our result demonstrates a novel activity of *A. sieboldianus* in addition to its previously reported antioxidant and hyperglycemia-suppressing properties [4,5]. We infer that inhibiting blood lactate elevation during performing high-intensity exercise by ingesting this plant as a dietary supplement could suppress production of reactive oxygen species (ROS), improve metabolic capability, and prevent an excessive stress response.

We examined the effect of *A. sieboldianus* ingestion at various levels of exercise intensity and confirmed that the plant extract decreased the blood lactate concentrations during and after the swimming test, which represented high-intensity exercise. Lactate accumulation in the blood depends on the balance between lactate production by muscle activity and lactate removal by the liver and other tissues [14]. The glycolytic system is a major source of energy for the active muscles during high-intensity exercise, and the lactate accumulates when production outstrips removal. When lactate accumulates in the working muscles, the muscle hydrogen ion concentration increases and the pH decreases; furthermore, activation of phosphofructokinase, a rate-controlling enzyme in the glycolytic system, decreases, inhibiting the re-synthesis of ATP [15]. Consequently, muscle strength, which is necessary for high-intensity exercise becomes difficult as lactate accumulation leads to physical fatigue. In fact, previous studies have revealed that accumulation of lactate in the blood decreases endurance exercise performance [16]. In the current study, ingestion of *A. sieboldianus* before exercise reduced the blood lactate concentrations observed after high-intensity exercises in comparison with ingestion of water demonstrating an anti-fatigue effect of this herbal supplement.

The antioxidant function of *A. sieboldianus* has been suggested to underlie its inhibition of blood lactate level elevation. Previous reports have demonstrated an increased level of ROS during high-intensity exercise [17]. In addition, the blood lactate and ROS concentrations generated during exercise have been reported to correlate [18]. Another study found that ingestion of an antioxidant substance inhibited the production of ROS [19]. Although the details of the mechanism behind the correlation between the blood lactate and ROS levels remain unclear, we hypothesize that the antioxidant function of *A. sieboldianus* suppresses the increase in the blood lactate concentrations during high-intensity exercise.

Next, regarding the depressive action on blood lactate levels due to *A. sieboldianus* intakes may be relate to the efficiency of oxygen use and thus facilitating ATP re-synthesis [20]. Aguilo *et al.* [20] investigated the effect of antioxidant supplementation in amateur sportsmen and reported that carbon dioxide (CO2) excretion was elevated during high-intensity exercise (lactate threshold: 4 mM). During the acidotic state following high-intensity exercise, CO2 is eliminated into the exhaled breath to compensate for the metabolic acidosis. Because antioxidants can promote CO2 excretion during exercise, they may assist in the respiratory compensation for the acidotic state even during high-intensity exercise, and this presumably results in inhibition of lactate accumulation. The plasma volume might also have had an effect. As the intensity of exercise increases, the plasma volume declines. This reduction affects the osmotic pressure, the concentrations of electrolytes, and even the lactate level of the blood. In other words, because water or ASS consumption prior to exercise inhibited the reduction in plasma volume, increases in the blood lactate concentration in the present study were attenuated compared with previous studies.

Finally, the suppression of the blood lactate concentration after ingestion of *A. sieboldianus* is likely to involve an anti-stress effect. The swimming tests used in our study subjected the rats to a great deal of psychological stress in addition to the exercise stress. In this study, the intake of ASS had no effect on the lactate and glucose concentrations during treadmill exercise. These findings suggest differences in the characteristics of the stressors involved in the two different types of exercise. In a study on exercise as an influence on stress hormones in horses, Alexander *et al.* reported that adrenocorticotropic hormone (ACTH), stress hormone, secretion during running exercise is more related to increases in the arginine vasopressin (AVP) than to corticotrophin-releasing hormone (CRH) levels [21]. In contrast, a study by Jiang *et al.* demonstrated increased secretion of CRH 10 min after a swimming exercise [22]. In this study, at least 5 sessions of treadmill habituation were performed before treadmill running test. Therefore, the relatively modest increase in the blood lactate concentration may have been due to the absence of psychological stress, as the subjects become accustomed to treadmill exercise. Furthermore, the treadmill running in this study was estimated to correspond to approximately 70% of VO_2max_, *i.e.*, mid-to-high-intensity exercise at just over the AT. However, this does not represent ultra-high-intensity exercise. This moderate intensity probably accounts for the relatively low blood lactate level produced. The swimming, by contrast, did not involve any practice analogous to the treadmill habituation and so presumably induced a greater stress reaction with substantial involvement of CRH in the ACTH secretion. Therefore, the current results suggest that ASS may be more effective against stress stimuli involving the secretion of CRH rather than AVP. A previous study in which Siberian ginseng (*A. senticosus* Harms), which, like *A. sieboldianus*, is a member of the *Araliaceae* family, was administered orally found an enhanced anti-stress effect attributed to activation of the noradrenaline system [23]. The *A. sieboldianus* used in our study may exert a similar anti-stress effect by suppressing the glycolytic mechanism induced by excessive sympathetic nervous system activation in response to physical and psychological stresses and thus inhibiting elevations of the blood lactate concentration.

## 5. Conclusions

We confirmed that administration of *A. sieboldianus* significantly decreases the blood lactate concentrations during and after high-intensity exercise relative to administration of water alone. This result suggests that ingestion of *A. sieboldianus* can help to alleviate fatigue during high-intensity exercise. In analyzing the mechanisms of this phenomenon, we assumed the potential involvement of all of the factors discussed above. Further investigation is necessary to determine whether all or only a single one of these factors are involved or whether the different mechanisms are related. It is also important to examine the other short-term effects of this herbal supplement as well as the effects of chronic administration in light of the results of this study.

## References

[1] American College of Sports Medicine Position Stand (1998). The recommended quantity and quality of exercise for developing and maintaining cardiorespiratory and muscular fitness, and flexibility in healthy adults. Med. Sci. Sports Exerc..

[2] Brug J., Lien N., Klepp K.I., van Lenthe F.J. (2010). Exploring overweight, obesity and their behavioural correlates among children and adolescents: Results from the Health-promotion through Obesity Prevention across Europe project. Public Health Nutr..

[3] Heller T., McCubbin J.A., Drum C., Peterson J. (2011). Physical activity and nutrition health promotion interventions: What is working for people with intellectual disabilities?. Intellect. Dev. Disabil..

[4] Tabuchi M., Tamura A., Matsuba S., Onodera J., Yamada N. (2004). Inhibitory effects of ukogi (*Acanthopanax sieboldianus*) leaves on postprandial blood glucose elevation in rats. J. Jpn. Soc. Nutr. Food Sci..

[5] Yamada N., Tamura A., Tabuchi M. (2003). Composition characteristics and antioxidative activity of ukogi (*Acanthopanax siebldianum*). Bull. Yamagata Prefect. Yonezawa Women’s Jr. Coll..

[6] Panossian A., Wagner H. (2005). Stimulating effect of adaptogens: An overview with particular reference to their efficacy following single dose administration. Phytother. Res..

[7] Fujikawa T., Yamaguchi A., Morita. I., Takeda H., Nishibe S. (1996). Protective effects of *Acanthopanax senticosus* Harms from Hokkaido and its components on gastric ulcer in restrained cold water stressed rats. Biol. Pharm. Bull..

[8] Zhang X.L., Ren F., Huang W., Ding R.T., Zhou Q.S., Liu X.W. (2011). Anti-fatigue activity of extracts of stem bark from *Acanthopanax senticosus*. Molecules.

[9] Norton K., Norton L., Sadgrove D. (2010). Position statement on physical activity and exercise intensity terminology. J. Sci. Med. Sport.

[10] Mazzeo R.S., Marshall P. (1998). Influence of plasma catecholamines on the lactate threshold during graded exercise. J. Appl. Physiol..

[11] Podolin D.A., Munger P.A., Mazzeo R.S. (1991). Plasma catecholamine and lactate response during graded exercise with varied glycogen conditions. J. Appl. Physiol..

[12] Chang H., Saito T., Ohiwa N., Tateoka M., Deocaris C.C., Fujikawa T., Soya H. (2007). Inhibitory effects of an orexin-2 receptor antagonist on orexin A- and stress-induced ACTH responses in conscious rats. Neurosci. Res..

[13] Chang H., Park J., Suk M., Lee H., Kang H., Choi K., Song W. (2009). Comparison of lactate threshold, glucose, and insulin levels between OLETF and LETO rats after all-out exercise. J. Sports Sci. Med..

[14] Stanley W.C., Gertz E.W., Wisneski J.A., Morris D.L., Neese R.A., Brooks G.A. (1985). Systemic lactate kinetics during graded exercise in man. Am. J. Physiol..

[15] Baker J.S., McCormick M.C., Robergs R.A. (2010). Interaction among skeletal muscle metabolic energy systems during intense exercise. J. Nutr. Metab..

[16] Sjödin B., Jacobs I. (1981). Onset of blood lactate accumulation and marathon running performance. Int. J. Sports Med..

[17] Viña J., Gimeno A., Sastre J., Desco C., Asensi M., Pallardó F.V., Cuesta A., Ferrero J.A., Terada L.S., Repine J.E. (2000). Mechanism of free radical production in exhaustive exercise in humans and rats; role of xanthine oxidase and protection by allopurinol. IUBMB Life.

[18] Sastre J., Asensi M., Gascó E., Pallardó F.V., Ferrero J.A., Furukawa T., Viña J. (1992). Exhaustive physical exercise causes oxidation of glutathione status in blood: Prevention by antioxidant administration. Am. J. Physiol..

[19] Zembron-Lacny A., Szyszka K., Sobanska B., Pakula R. (2006). Prooxidant-antioxidant equilibrium in rowers: Effect of a single dose of vitamin E. J. Sports Med. Phys. Fitness.

[20] Aguiló A., Tauler P., Sureda A., Cases N., Tur J., Pons A. (2007). Antioxidant diet upplementation enhances aerobic performance in amateur sportsmen. J. Sports Sci..

[21] Alexander S.L., Irvine C.H., Ellis M.J. (1991). The effect of acute exercise on the secretion of corticotropin-releasing factor, arginine vasopressin, and adrenocorticotropin as measured in pituitary venous blood from the horse. Endocrinology.

[22] Jiang Y.-Q., Kawashima H., Iwasaki Y., Uchida K., Sugimoto K., Itoi K. (2004). Differential effects of forced swim-stress on the corticotropin-releasing hormone and vasopressin gene transcription in the parvocellular division of the paraventricular nucleus of rat hypothalamus. Neurosci. Lett..

[23] Soya H., Deocaris C.C., Yamaguchi K., Ohiwa N., Saito T., Nishijima T., Kato M., Tateoka M., Matsui T., Okamoto M. (2008). Extract from *Acanthopanax senticosus* harms (Siberian ginseng) activates NTS and SON/PVN in the rat brain. Biosci. Biotechnol. Biochem..

